# Direct Purification of Pectinase from Mango (*Mangifera Indica Cv. Chokanan*) Peel Using a PEG/Salt-Based Aqueous Two Phase System

**DOI:** 10.3390/molecules16108419

**Published:** 2011-10-10

**Authors:** Amid Mehrnoush, Md. Zaidul Islam Sarker, Shuhaimi Mustafa, Abdul Manap Mohd Yazid

**Affiliations:** 1 Department of Food Technology, Faculty of Food Science and Technology, Universiti Putra Malaysia, 43400 UPM Serdang, Selangor, Malaysia; 2 Department of Pharmaceutical Technology, Faculty of Pharmacy, International Islamic University Malaysia, Kuantan Campus, Bandar Indera Mahkota, 25200 Kuantan, Pahang, Malaysia; E-Mail: zaidul@iium.edu.my (M.Z.I.S.); 3 Department of Microbiology, Faculty of Biotechnology and Biomolecular Science, Universiti Putra Malaysia, 43400 UPM Serdang, Selangor, Malaysia; E-Mail: Shuhaimi@biotech.upm.edu.my (S.M.)

**Keywords:** purification, pectinase, Mango peel, aqueous two-phase system (ATPS), yield

## Abstract

An Aqueous Two-Phase System (ATPS) was employed for the first time for the separation and purification of pectinase from mango (*Mangifera Indica Cv. Chokanan*) peel. The effects of different parameters such as molecular weight of the polymer (polyethylene glycol, 2,000–10,000), potassium phosphate composition (12–20%, w/w), system pH (6–9), and addition of different concentrations of neutral salts (0–8%, w/w) on partition behavior of pectinase were investigated. The partition coefficient of the enzyme was decreased by increasing the PEG molecular weight. Additionally, the phase composition showed a significant effect on purification factor and yield of the enzyme. Optimum conditions for purification of pectinase from mango peel were achieved in a 14% PEG 4000-14% potassium phosphate system using 3% (w/w) NaCl addition at pH 7.0. Based on this system, the purification factor of pectinase was increased to 13.2 with a high yield of (97.6%). Thus, this study proves that ATPS can be an inexpensive and effective method for partitioning of pectinase from mango peel.

## 1. Introduction

Mango (*Mangifera Indica Cv. Chokanan*) is one of the most important and popular commercial tropical fruits in the World. In the processing of mango products like mango pulp and amchur (dried, powdered unripe mango), mango peel is a major byproduct [1] of mango juice industry. There are so many mango juice industries are available in Malaysia as the mango juice is very popular and common juice in the country. However, despite the presence of a lot of useful enzymes such as pectinase, protease, amylase and xylanase in mango peel, this potential source is currently not being commercially utilized in any way and the peel ends up being a waste product, thus contributing to pollution, and with the industry incurring in a high cost for its waste treatment [2]. Mango peel can be used as a valuable, economic and abundant media source for the commercial production of the natural enzymes such as pectinase [3]. Pectinases are a group of enzymes, which can catalyze the breakdown of pectin bounds in plant cells [4]. They are widely used in various industries for cloud point stabilization in fruit and vegetable nectars, fermentation of cocoa beans, and clarification of fruit juices in the food industry [5]. More recently, pectinase has been used in textiles for degumming of fiber crops, in waste treatment, and the paper industries [6].

The current commercial sources of pectinase comprise a wide variety of bacteria (such as *B. polymyxa*, *Erwinia spp*, *E. carotovora*, *Ps. syringae*), some yeasts like *Kl. fragilis* and *Kl. marxianus* and filamentous fungi. Among the filamentous fungi *Aspergillus niger* is the most popular source for production of pectinase. Submerged and solid state fermentation are two types of fermentation technique employed to produce enzymes from microorganisms. Different methods of purification of the desired products are used, including, in the case of pectinase purification. Conventional methods such as gel filtration, hydrophobic interaction or anion exchange chromatography [7].

The conventional purification processes are multistep, discontinuous, as well as time and labor intensive, which all add to the cost and also could cause loss of product yield and quality [8]. The so-called Aqueous Two-Phase System (ATPS), made up of two polymers or one polymer and one salt has become an attractive method in the production of industrial enzymes compared to conventional purification methods [9], Due to the fact that the scale-up of ATPS is easy, the use of this method is desirable. Also, separation times are short, and the technique is non-toxic [10]. In such a system, manipulation of important parameters can help to achieve a desirable protein partitioning.

Currently, there is no literature on the purification of pectinase from mango (*Mangifera Indica Cv. Chokanan*) peel using ATPS. The main challenge in using ATPS is that the purification of macromolecules comes at a high cost due to the number of polymers used. In order to reduce the cost, the expensive polymer (dextran) is substituted with a lower cost polymer (PEG). In the present study, PEG/salt ATPS was chosen to purify pectinase and important parameters affecting the partitioning and recovery of the enzyme such as molecular weight of the polymer, phase composition, pH and NaCl addition were investigated.

## 2. Result and Discussion

### 2.1. Effect of PEG Molecular Weight on Partitioning of Pectinase

First the effect of PEG molecular weight (2,000–10,000 g/mol) on partitioning and yield of pectinase from mango peel was investigated. The partition coefficient, purification factor and yield of the enzyme decreased as the PEG molecular weight increased (Table 1). In general, the volume to accommodate the target enzyme in the top phase (PEG-rich phase) was decreased when the polymer molecular weight was increased and thus the target enzyme tends to be partitioned in the bottom phase as a result of a decrease in the pectinase partition coefficient [11]. The yield of the enzyme was increased with 4,000 g/mol PEG and it seems that in this case there is suitable available free volume to accommodate pectinase in the top phase, while a decrease in pectinase yield at PEG 2,000 g/mol was observed because low molecular mass is unsuitable for sufficient partitioning of pectinase as contaminants could also be transferred to the top phase with the target enzyme and as a result decrease the enzyme yield. As considered earlier, the yield of pectinase was decreased at PEG high molecular weight, possibly due to increased partitioning of the enzyme to the bottom phase. Also, decreasing of the purification factor of pectinase at high PEG molecular weight is the result of additional partitioning of pectinase to the bottom phase compared to other proteins. Based on the results, 4,000 g/mol PEG was chosen as the best PEG molecular weight for subsequent studies.

### 2.2. Effect of Potassium Phosphate Concentration on Partitioning of Pectinase

The influence of different concentrations of potassium phosphate ranging from 12–20% (w/w) on the partition behavior of pectinase from mango peel was investigated. The partition coefficient of pectinase was increased by increasing the potassium phosphate concentration from 12 to 16 % (w/w) (Table 2). This could be due to the salting-out effect of potassium phosphate which leads to an increase of pectinase partitioning into the top phase [12]. On the other hand, the partition coefficient (K) and yield (Y%) of pectinase were decreased at higher salt concentrations because when the potassium phosphate concentration increased, the salting-out effect of salt is also increased. This takes place in the bottom phase, resulting in contaminating proteins and target enzyme being moved to the top or interphase, thus reducing the purity of the enzyme The data shows that an increase in salt concentration to 20% (w/w) causes a decrease in the partition coefficient of the protein, resulting in a decrease in the purification factor, and yield [13]. As shown in Table 2, the best partition behavior was achieved at 14% (w/w) of potassium phosphate concentration and thus the 14% PEG 4000/14% potassium phosphate combination was selected for the next experiments.

### 2.3. Effect of System pH on Partitioning of Pectinase

**Figure 1:**
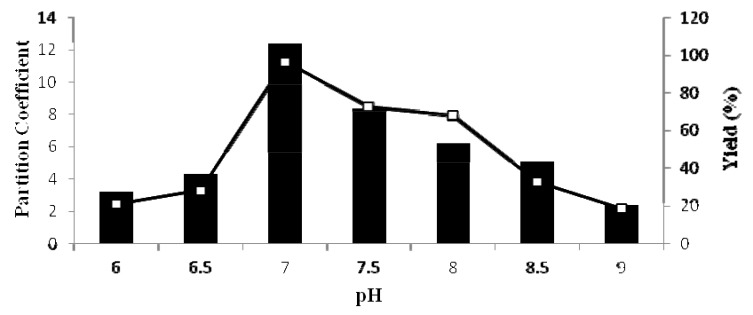
The pH of ATPS was varied between 6.0 and 9.0. The pectinase partition coefficient (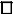) and yield (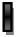) were calculated using Equation 1 and Equation 4, respectively.

The influence of pH on partitioning and yield of pectinase in phase system is shown in Figure 1. As shown in the figure, an average yield of 96.7% was achieved in pH range 6–9. The yield and pectinase activity were significantly decreased when attempted at pH values of less than 6.5, and most of the pectinase was partitioned in the bottom phase. This change of partition behavior of pectinase is caused by the effect of pectinase charge. The ionizable groups and surface charge of the protein could be influenced by the pH of the system [14]. When the pH of the system is changed, enzyme will be separated because of the net charge of protein and surface properties rather than the charge [15]. The isoelectric point of pectinase is about 6.1, and at pH 6.5 the pectinase attracts a small negative charge and partitioning is based on surface properties rather than the net charge. The pectinase also has negative charge at pH above 7–8, while PEG tends to act with positive charge. Therefore, this interaction at pH 7.0 improved the partitioning of pectinase to the top phase as the highest partition coefficient of the enzyme was obtained at pH 7.0 (12.4). In addition, the yield at pH 7.0 was the highest (96.7%). Thus, the system with pH 7.0 was chosen for future study.

### 2.4. Effect of NaCl on Partitioning of Pectinase

**Figure 2:**
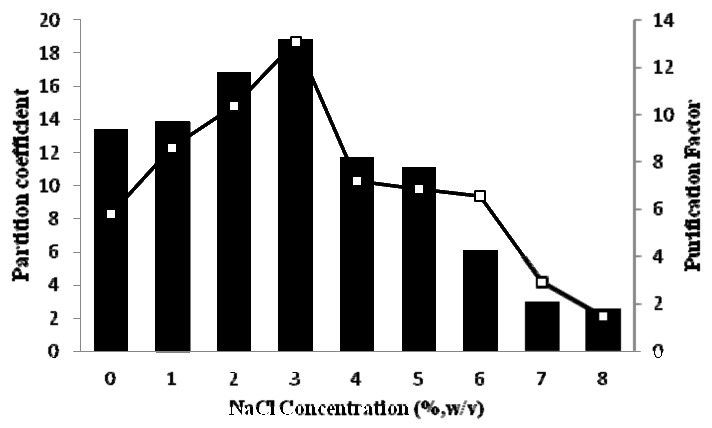
All the scouting experiments were carried out with 14% PEG 4000/14% potassium phosphate at pH 7.0. The partition coefficient (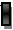) and purification factor (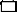) were calculated as a function of the NaCl concentration.

The effect of different concentrations of NaCl [0–8% (w/v)] was studied for 14% PEG 4000/14% potassium phosphate at pH 7.0 and the result is shown in Figure 2. The addition of NaCl to the ATPS system leads to an increase in the hydrophobicity difference between the two phases and enhances partitioning of hydrophobic proteins to the top phase. Increasing the NaCl concentration also promotes the resolution of the system [16], meaning that the partition coefficient between the two phases increases with NaCl concentration. The addition of NaCl to the ATPS could have an effect on water structure and provide an improved interaction between the hydrophobic chain of PEG and the hydrophobic surface area of pectinase [17]. As shown in Figure 2, The partition coefficient (18.7) and purification factor (13.2) of pectinase were markedly increased in the presence of 3% (w/v) of NaCl, while the higher concentration of NaCl had a negative effect on the partition behavior of pectinase because the unequal partitioning of the natural salt between two phases could affect the potential chemical of the solute.

### 2.5. SDS-PAGE Analysis of Purified Pectinase from ATPS

**Figure 3:**
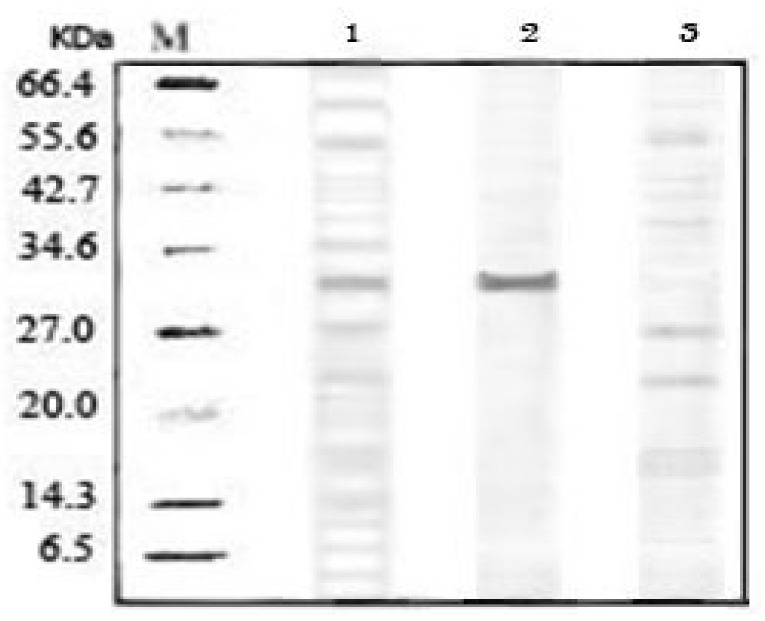
The purity of the partitioned pectinase was assessed by SDS-PAGE analysis. M = protein molecular markers (6.5-212 kDa); lane 1 = crude feedstock; lane 2 = ATPS top phase; lane 3 = ATPS bottom phase.

The purity of pectinase was investigated with an SDS-PAGE Laemmli [18] and is shown in Figure 3. Lane 1 shows the initial feedstock with a range of bands representing impurities. Lane 3 corresponds to the sample from the bottom phase, which indicates lesser and fainter bands than crude feedstock. The sample taken from the top phase with the highest pectinase activity and purity is shown as a dark band with molecular weight 31 kDa. Therefore, this SDS-PAGE result shows that the purification technique employed in this study gives maximal recovery of pectinase from mango peel.

## 3. Experimental Section

### 3.1. Materials

Mango fruits (*Mangifera Indica Cv. Chokanan*) used in this study were purchased from a local market in Selangor, Malaysia in slightly under-ripened commercial maturity stage with a Brix value of 14. The fruits were washed with double-distilled water and the peel was cut into small cubes (3 mm × 3 mm × 1 mm) and stored at 4 °C for further study.

### 3.2. Chemicals

All chemicals and reagents used were analytical grade. Pectin from citrus fruits, bovine serum albumin (BSA), 3,5-dinitrosalicylic acid (DNS) and Bradford reagent were supplied by Sigma Chemical Co. (St. Louis, MO, USA). Polyethylene glycol (PEG), sodium dodeycel sulfate (SDS), trichloroacetic acid (TCA, 99%), disodium hydrogen phosphate anhydrous, sodium hydrogen phosphate monohydrate, di-potassium hydrogen phosphate and potassium di-hydrogen phosphate, were purchased from Merck (Darmstadt, Germany).

### 3.3. Enzyme Extraction

The small pieces of mango peel were blended with a commercial laboratory blender 32BL79 (Dynamic Corporation of America,Torrington, CT, USA) in cold extraction buffer (50 mM sodium hydrogen phosphate, pH 7.5) in 1:2 ratio for 4 min at high speed. Following this, the homogenate was filtered through cheesecloth and then filtered solution was centrifuged at 6,000 g for 10 min at 4 °C. The crude enzyme extract (supernatant) was employed for subsequent experiments [19] 

### 3.4. Aqueous Two Phase System

An appropriate amount of PEG, potassium phosphate and double-distilled water was weighed and added to the crude enzyme to obtain a final mass of 100% (w/w) system. The weight of the crude extract was 25% (w/w) during all experimental stages. The contents were mixed gently for equilibration and then centrifuged to achieve the phase separation at 4,000 rpm for 10 min. Using a pipette, the upper phase was removed while the lower phase was then collected. Samples of each phase were analysed for enzyme activity and protein concentration. To decrease the possibility of PEG and KH_2_PO4 interferences the system controls were obtained by adding 20% (w/w) double-distilled water rather than enzyme. All partition experiments were conducted in triplicate at 25 ± 1 °C.

### 3.5. Pectinas Activity Assay

The pectinase activity was determined according to the procedure of Miller [20] with some modification. An aliquot of suitably diluted enzyme (0.5 mL) was added to 1% (w/w) of citrus pectin in 0.5 M acetate buffer at pH 4.3 in a test tube. The mixture was incubated at 50 °C for 30 min in a water bath. After incubation, 3,5-dinitrosalicylic acid (DNS, 3 mL) were added to the solution to stop the reaction and the tube was kept in boiling water for 5 min. A spectrophotometer (BioMate^TM^-3, Thermo Scientific, Alpha Numerix, Woodfield Dr, Webster, NY, USA) was used to determine the pectinase activity at 575 nm, and galactouronic acid was used as a standard to measure the recuing sugar release. The results were carried out as a mean of three readings with as estimated error of ±10%.

### 3.6. Protein Concentration Determination

The protein contents of samples were determined using dye binding method as described by Bradford [21] and BSA was used as standard.

### 3.7. Determination of Partition Coefficient, Purification Factor and Yield of Enzyme

The partition coefficient (K) of pectinase was determined by the ratio of pectinase activity in two phases:

K = *A_T_* / *A_B_* (1)

where A_T_ is activity of pectinase in top phase and A_B_ is activity of the enzyme in bottom phase. The specific activity of the enzyme was determined by taking the ratio of total activity of serine protease and dividing it by total protein of serine protease [22]:

Specific activity (U/mg) = Total activity (U) / Total protein (mg)(2)

The ratio of specific pectinase activity in the top phase to the initial specific activity of the enzyme was employed for the calculation of the purification factor of the top phase (P_FT_):

P_FT_ = *Specific activity of top phase sample* / *Initial Specific activity* (3)

Yield of pectinase was estimated using:

Y_T_ (%) = *100*/ *1* + *(1/ [V_R_]·[K])* (4)

where V_R_ is volume ratio top phase to bottom phase [23].

### 3.8. Sodium Dodecyl Sulfate-Polyacrylamide Gel Electrophoresis (SDS-PAGE)

The purity of pectinase after purification by ATPS was investigated using SDS-PAGE according to the method of Laemmli [18]. Protein bands were observed using the silver staining method [24].

## 4. Conclusion

The partitioning of pectinase from mango (*Mangifera Indica Cv. Chokanan*) peel in PEG and potassium phosphate ATPS was successfully performed. The findings in this study showed that this purification procedure could be employed as an efficient and attractive method for the partitioning and recovery of pectinase. The overall optimum region resulted in suitable pectinase purification results (a purification factor of 13.2 and a yield of 96.7%) using 14% (w/w) PEG 4000 and 14% (w/w) potassium phosphate at pH 7.0 with addition of 3% (w/v) of NaCl. Distinctively, the results show that the PEG molecular weight, salt concentration, pH and NaCl addition should be considered as important parameters for pectinase purification. The findings indicate that the ATPS methid could be a beneficial, attractive and economical technique for separation and recovery of pectinase.

## Figures and Tables

**Table 1:** Effect of PEG molecular weight on partition coefficient, yield and purification factor of pectinase.

PEG Type ^a^	Partition coefficient (K)	Yield (%)	Purification factor (P_FT_)
2,000	4.3	48.3	6.3
4,000	5.8	69.2	8.7
6,000	3.2	22.4	5.2
8,000	1.4	33.5	3.5
10,000	0.9	12.5	4.8

^a^ 14% (w/w) PEG + 14% (w/w) potassium phosphate.

**Table 2:** Effect of potassium phosphate concentration on partition coefficient, yield and purification factor of pectinase.

Concentration (%, w/w) ^a^	Partition coefficient (K)	Yield (Y%)	Purification factor (P_FT_)
PEG 4000 (14%)/ Potassium phosphate (12–20%,w/w)			
12	2.2	67.3	6.9
14	8.3	73.8	9.4
16	5.7	54.2	7.2
18	2.1	33.2	4.4
20	0.5	23.4	3.2

^a^ 14% (w/w) PEG+ 12–20% (w/w) potassium phosphate.
